# Madeira Wine Volatile Profile. A Platform to Establish Madeira Wine Aroma Descriptors

**DOI:** 10.3390/molecules24173028

**Published:** 2019-08-21

**Authors:** Rosa Perestrelo, Catarina Silva, José S. Câmara

**Affiliations:** 1 CQM—Centro de Química da Madeira, Universidade da Madeira, Campus da Penteada, 9020-105 Funchal, Portugal; 2 Departamento de Química, Faculdade de Ciências Exatas e Engenharia, Universidade da Madeira, Campus da Penteada, 9020-105 Funchal, Portugal

**Keywords:** wine, VOCs, potential odorants, HS–SPME, GC–qMS

## Abstract

In the present study we aimed to investigate the volatile organic compounds (VOCs) that may potentially be responsible for specific descriptors of Madeira wine providing details about Madeira wine aroma notes at molecular level. Moreover, the wine aroma profile, based on the obtained data, will be a starting point to evaluate the impact of grape variety (Malvasia, Bual, Sercial, Verdelho and Tinta Negra), type (sweet, medium sweet, dry and medium dry), and age (from 3 to 20 years old) on Madeira wine sensorial properties. Firstly, a comprehensive and in-depth Madeira wine volatile profiling was carried out using headspace solid-phase microextraction combined with gas chromatography-mass spectrometry (HS–SPME/GC–qMS). Secondly, a relation among the varietal, fermentative and aging aroma compounds, and their aroma descriptors with the Madeira wine sensorial properties was assessed. A total of 82 VOCs, belonging to different chemical families were identified, namely 21 esters, 13 higher alcohols, ten terpenic compounds, nine fatty acids, seven furanic compounds, seven norisoprenoids, six lactones, four acetals, four volatile phenols and one sulphur compound. From a sensorial point of view, during the aging process the wine lost its freshness and fruitiness odor related to the presence of some varietal and fermentative compounds, whereas other descriptors such as caramel, dried fruits, spicy, toasty and woody, arose during ageing. The Maillard reaction and diffusion from the oak were the most important pathways related with these descriptors. A relationship-based approach was used to explore the impact of grape variety, wine type, and age on Madeira wine sensorial properties based on shared number of VOCs and their odors.

## 1. Introduction

Madeira wine is a fortified Portuguese wine produced in Madeira Island over the last centuries playing an important role in the Island economy. The specific characteristics of Madeira wine arise from a set of specific conditions including the terroir, unique grape varieties and the singular winemaking process. The fermentation process is stopped by the addition of natural grape spirit in order to obtain an ethanol content of 18–22% (*v*/*v*). Some wines are submitted to an aging process in oak casks, in cellars, at temperatures up to 30 °C, and humidity levels between 70 and 75%, while the majority of wines are submitted to a baking process, i.e., the wine is placed in large coated vats and the temperature is slowly increased at about 5 °C per day, and maintained at 45–50 °C for at least 3 months. After this treatment, the wine is allowed to undergo a maturation process in oak casks for a minimum of 3 years [1,2].

Wine volatile composition plays an important role in wine quality since it promotes several sensations during wine consumption, odors (due to molecules that can bind olfactory receptors) and can affect flavor (combination of aroma and taste) in mouth retro-nasally, that lead to consumer acceptance or rejection. The wine aroma consists of a combination of several hundred of different volatile organic compounds (VOCs), most of which are present in trace amounts (usually at μg/L or ng/L level) [3]. Nevertheless, the presence of a molecule at a concentration above its odor threshold (OT), is sufficient to provide a characteristic product aroma (impact odorant). Nevertheless, even when present at concentrations below their OTs, may contribute to the overall wine aroma, as a result of the interactions with other molecules [4]. 

Different extraction techniques, such as solid phase extraction (SPE) [5,6,7,8,9] and liquid-liquid extraction [5,10,11,12,13] have been applied on the establishment of volatile profile of Madeira wine. However, most of these approaches present several disadvantages, such as time- and labor-intensive, large solvent and sample amount, which can lead to analyze losses and a reduction in sensitivity. Currently, the trend in the analyze of VOCs is more focused in the use of miniaturized sample preparation, increasing of efficiency of analysis, no solvent techniques, such as solid-phase microextraction (SPME) [7,10,14,15], stir bar sorptive extraction (SBSE) [14] and microextraction by packed sorbents (MEPS) [7] followed by gas chromatography-mass spectrometry (GC-MS) process have been used for that purpose. GC-MS is user-friendly, fast, selective and very sensitive method to establish the volatile signature of several food matrices. In addition, it was also equipped with powerful data systems that are used not only to control and acquire data from the GC and MS, but also to identify flavor components by automated matching against reference libraries of spectra of known odorants [16].

The Madeira wines volatile composition has been topic of several studies, as the data obtained has been useful in the elucidation of basic flavor chemistry. Enormous efforts were focused on the topic of varietal (e.g., terpenoids, norisoprenoids) [14,17,18], pre-fermentative (e.g., C_6_ alcohols and aldehydes) [11], fermentative (e.g., alcohols, esters, acids, carbonyl compounds) [5,8,14], and finally aging aroma compounds (e.g., volatiles extracted from oak, like volatile phenols, lactones) [5,8,15,19]. Few studies have focused on the establishment of potential impact odorants, which could contribute individually to the Madeira wine aroma [6]. Campo et al. [6] build a hierarchical list of the odorants using gas chromatography-olfactometry (GC-O) that express the aroma of Madeira wines. The GC–O profile of Madeira wines lacks on varietal compounds (e.g., terpenoids, cystein-derived thiols), is rich in sotolon, phenylacetaldehyde, (*Z*)-whiskey lactone and of some volatile phenols (e.g., guaiacol, 4-vinylguaiacol, *m*-cresol). Madeira wines contain a huge number of intense odorants not identified which were not even detected in the corresponding young wines [6]. Sotolon has also been previously reported as powerful odorant, responsible for burnt, curry, honey, nutty, spicy, walnut odors of Madeira wines, depending on their concentration and enantiomeric distribution [12,20]. 

This research aimed to provide details about Madeira wine aroma notes at molecular level, as it can be useful to explain its peculiar aroma. Moreover, the wine aroma profile is a natural starting point for a systematic search for principles to evaluate the impact of grape variety, type, and age on Madeira wine sensorial properties. Therefore, Madeira wines from different varieties (Malvasia, Bual, Sercial, Verdelho and Tinta Negra), types (sweet, medium sweet, dry and medium dry), and ages (from 3 to 20 years old) were analyzed. Firstly, in-depth Madeira wine volatile profiling (e.g., terpenic compounds, norisoprenoids, sulphur compound, alcohols, esters, lactones, furanic compounds, acetals) was carried out using HS–SPME/GC–qMS. Secondly, a relation among the varietal, fermentative and aging aroma compounds, and their aroma descriptors with the Madeira wine sensorial properties was performed. A relationship-based approach was used to explore the impact of grape variety, wine type, and age on Madeira wine sensorial properties based on shared number of VOCs and their odor descriptors. 

## 2. Results and Discussion

HS–SPME/GC–qMS methodology was used to establish the Madeira wine volatile profiling, as a sensitive technique to explain the unique aroma descriptors of Madeira wines. Considering the five grape varieties under study, a set of 82 VOCs (Table 1), namely 21 esters, 13 higher alcohols, ten terpenic compounds, nine fatty acids, seven norisoprenoids, seven furanic compounds, six lactones, four acetals, four volatile phenols and one sulphur compound (Tables S1 and S2), have been identified by matching the obtained mass spectra with the reference compounds spectra in NIST Mass Spectral Search Program with a resemblance percentage above 80% and by comparison of the KIs calculated (KI_calc_) with the values reported in the literature (KI_lit_) for polyethylene glycol (or equivalent) column. 

A range between 0 and 35 (|KI_calc_ − KI_lit_|) was obtained for KI_cal_ compared to the KI_lit_ reported in the literature for GC with polyethylene glycol GC column or equivalent. This difference in KI is acceptable (<5%) taking into account that the literature data is obtained from a large range of GC stationary phases (several commercial GC columns are composed of polyethylene glycol or equivalent stationary phases). The relative concentration of each VOC and their relative standard deviation (%RSD) in dry/medium dry and sweet/medium sweet are available as Supplementary Material on Tables S1 and S2, respectively. Sixty-nine VOCs were common in all wine samples analyzed, namely seven terpenic compounds, six norisoprenoids, 13 alcohols, 21 esters, 9 acids, four acetals, seven furanic compounds and two volatile phenols (Tables S1 and S2). 

The number of identified VOCs ranged from 77 to 79 for Malvasia wines, from 78 to 79 for Bual wines, from 76 to 80 for Sercial wines, and from 77 to 80 for Verdelho wines, for young and old wines, respectively. For Tinta Negra variety, different types of wines were considered. It was observed that the number of VOCs ranged from 77 to 79 for dry, from 80 to 82 for medium dry, from 76 to 80 for sweet, and from 79 to 81 for medium sweet, for young and old wines, respectively. 

### 2.1. Potential Impact Odorants of Madeira Wines

As observed in Table 2, young (3 to 5 years old) Madeira wines are characterized by freshness and fruitiness descriptors (e.g., citrus, floral, fruity), whereas the old (10 to 20 years old) Madeira wines are characterized by caramel, dried fruits, spice, toast and wood notes based on sensory analysis [6,46]. The information reported in Table 2 was obtained by a panelist of eleven expert judges belonging to different Madeira wine companies, such Madeira Wine Company, Barbeito, Borges, Justinos, and some employees from Instituto do Vinho Madeira, and also supported on previous studies [6,46].

**Figure 1:**
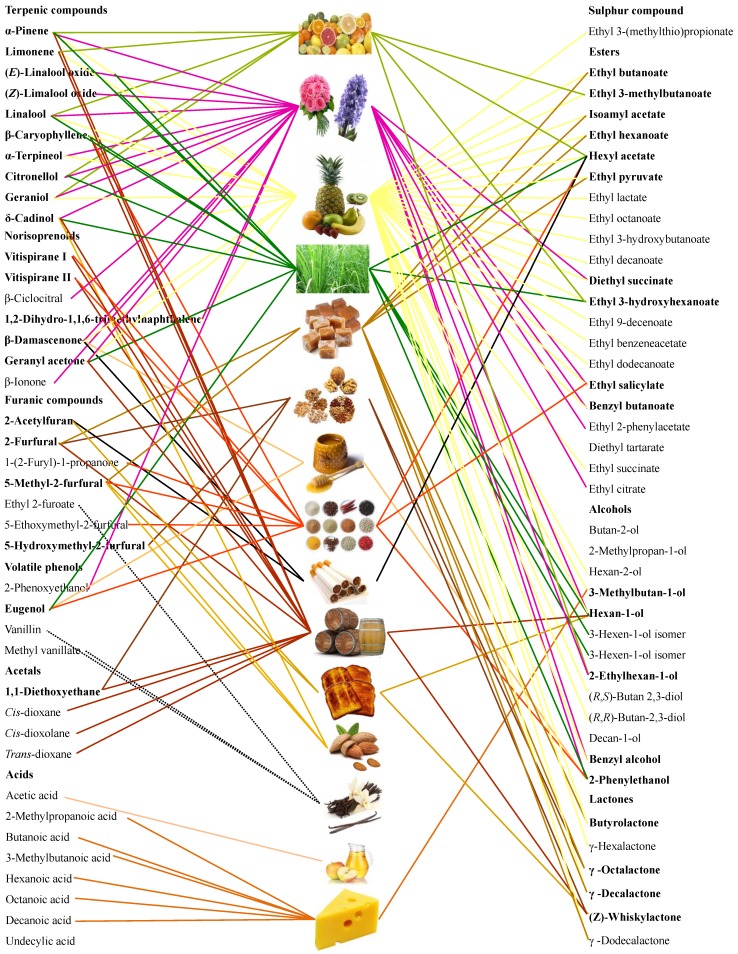
Madeira wine aroma notes (middle column), together with the chemical families (82 VOCs) that explain its aroma notes (left and right columns). Volatile compounds are shown in boldface if shared at least two or more aroma odors.

A relationship-based approach consisting of two different nodes was built: (i) 15 Madeira wine aroma notes, and (ii) 82 VOCs that are known to explain each of these aroma notes (Figure 1). 

**Figure 2:**
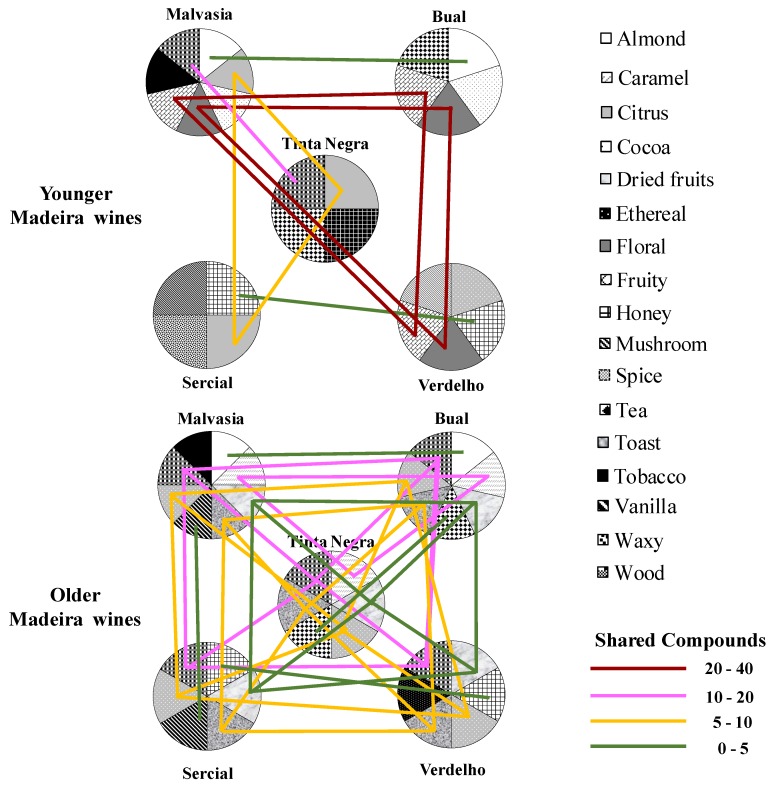
Aroma system. Each color represents an aroma note. The color line corresponds to the number of shared VOCs.

The concentration and OT of each VOC is necessary to determine its contribution to overall Madeira wine aroma. In the current research, a semi-quantification was performed in order to establish a potential relationship between Madeira wines profiling and their odor descriptors with wines sensorial evaluation. As observed in Figure 1, different aroma notes were found for the same VOC, which could be influenced by odor the intensity evaluation, as well as VOCs concentration and nature of matrix analyzed. So, the resulting aroma relationship-based approach is too complex to achieve more information [6,11,37,38,39,40,41,42,43,44,45]. A projection of this relationship-based approach is the aroma system (Figure 2), in which two nodes (Madeira wine aroma notes) are linked if they share at least one aroma note. The color line represents the number of shared compounds.

According to the obtained results, the grape variety seems to have a great impact in the sensorial properties of young Madeira wines, among several other parameters (e.g., vinification procedure). Specific aroma notes are linked to grape variety, as for example Malvasia and Bual grapes, used to produce sweet and medium sweet wines characterized by almond and cocoa odors. Dry and medium dry Madeira wines, obtained from Sercial and Verdelho grapes, are characterized by mushroom and honey notes. These grape varieties specific notes are shared, on average, by three VOCs. However, few aroma notes are connected between these grape varieties. Malvasia, Bual and Verdelho grapes are connected by flower and fruit odors and are shared, on average, by 31 VOCs, whereas the citrus odors linked to Malvasia, Tinta Negra and Sercial are shared by nine VOCs. In terms of Madeira aroma notes, young wines from Malvasia and Bual grapes are the most complex, contrarily to the observed for Tinta Negra, Sercial and Verdelho (Figure 2). For the oldest wines, it was observed that several aroma notes (e.g., dried fruit, spice, toast, wood) were present in all varieties under study. On average, eight VOCs that may explain these notes were shared by these varieties. Figure 2 shows that oldest wines, from the five varieties, presented higher aroma similarity than in youngest ones, which suggest the powerful impact of aging process on Madeira wine aroma.

#### 2.1.1. Young Madeira Wines

Taking into account the OTs [6,31,32,33,34,35,36] and odor descriptor [6,11,37,38,39,40,41,42,43,44,45] reported in Table 1, as well as the relative concentration of VOCs (Tables S1 and S2), the citrus odor characteristic of Malvasia, Sercial and Tinta Negra wines (Table 2) could be explain by the presence of some terpenic compounds, such as α-pinene, limonene, linalool, citronellol, geraniol, and some esters, like hexyl acetate, ethyl 3-methylbutanoate and ethyl 3-hydroxyhexanoate, and 2-ethylhexan-1-ol (Table 3). All these varietal and fermentative compounds are present in Malvasia, Sercial and Tinta Negra wines at relative concentrations higher than their respective OT.

α-Pinene, linalool, citronellol, geraniol, β-cyclocitral, 1,2-dihydro-1,1,6-trimethylnaphthalene (TDN), β-damascenone, geranyl acetone, β-ionone, 1-hexanol and 2-phenylethyl alcohol are some varietal aroma compounds that could explain the floral odor related to Malvasia, Bual and Verdelho young wines (Table 3). By the other hand, linalool oxide and α-terpineol cannot explained the floral odors, since they are present in Malvasia, Bual and Verdelho wines at relative concentrations lower than their OT. Generally, the relative concentration of these varietal VOCs (e.g., α-pinene, linalool, citronellol) decreased during aging process (Tables S1 and S2), which could explain the absence of these odors in old wines. Some varietal compounds, linalool [6], β-damascenone [10] and TDN [47] have been reported as important odorants related to violet, exotic fruit and/or exotic floral descriptors of young wines. The waxy odor of Sercial young wine could be explained by the presence of some terpenic compounds (e.g., geraniol, geranyl acetone) and esters (e.g., ethyl octanoate, ethyl decanoate), since their relative concentration decreased slightly during aging process and present low OTs (Table 1). Finally, the almond odor of Malvasia and Bual young wines could be explained by the presence of δ-cadinol.

#### 2.1.2. Old Madeira Wines

**Figure 3:**
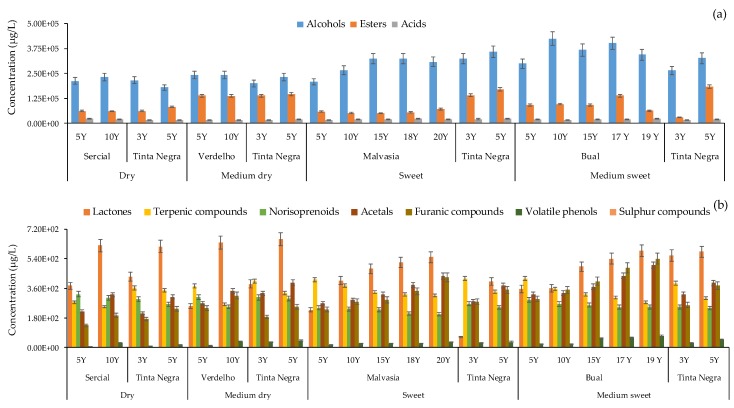
Total relative concentration (μg/L) of major (**a**) and minor (**b**) chemical families identified in Madeira wine.

The caramel descriptor characteristic of older Malvasia, Bual and Tinta Negra wines suggests the presence of some esters (e.g., ethyl butanoate, ethyl hexanoate, ethyl pyruvate), furans (e.g., 2-furfural, 5-methyl-2-furfural), and some lactones (e.g., γ-butyrolactone, γ-octalactone, (*Z*)-whiskylactone). The relative concentration of furans and lactones increased during Madeira wines aging (Figure 3b). Nevertheless, 2-furfural (OT = 14,100 µg/L), 5-methyl-2-furfural (20,000 µg/L), and γ-butyrolactone (OT = 35,000 µg/L) could not be used to explain the caramel descriptor since their relative concentration (Tables S1 and S2) was lower than their respective OTs (Table 1). Campo et al. [6] reported that furfural, 5-methylfurfural, 5-hydroxymethylfurfural and 5-ethoxymethylfurfural were not detected in the GC–O assays, in spite of the fact that these furanic compounds are quantitatively important, are not relevant to the aroma of Madeira wine. Moreover, phenylacetaldehyde, sotolon, (*Z*)-whiskylactone and some volatile phenols from wood are important odor active compounds in Madeira wines [6]. In the current study, from these three VOCs, only (*Z*)-whiskylactone was detected.

Thus, based on the OTs, ethyl butanoate, γ-octalactone and (*Z*)-whiskylactone could be the VOCs responsible for the caramel descriptor characteristic of older Malvasia, Bual and Tinta Negra wines, since their relative concentrations (Tables S1 and S2) were higher than their OTs. The ethereal descriptor characteristic of Verdelho wines suggests the presence of ethyl lactate and ethyl pyruvate. Perhaps, these two VOCs were also presented in all Madeira wines analyzed, the relative concentration of ethyl pyruvate in Verdelho wines (on average 3.54 µg/L) was higher than the remaining Madeira wines (on average 1.49 µg/L).

The presence of hexyl acetate, 2-phenyethyl alcohol, 5-(ethoxymethyl) furfural and eugenol could explain the spicy notes characteristic of old Madeira wines, since their relative concentration was higher than their respective OTs (Table 1). Other VOCs that could explain the spicy notes were ethyl pyruvate and ethyl salicylate, however no information related to their OTs is available. 2-Acetylfuran, 5-methyl-2-furfural and (*Z*)-whiskey lactone could explain the toast odor, as their relative concentration increased remarkably during aging process (Tables S1 and S2). Nevertheless, taking into account the relative concentration and OTs, (*Z*)-whiskey lactone is the potential odorant responsible for the toast notes characteristic of older Madeira wines. The vanilla odor related to Malvasia and Sercial wines could be explained by the presence of ethyl 2-furoate, vanillin and methyl vanillate, since a remarkably increase on relative concentration was observed for vanillin and ethyl vanillate during aging process. Vanillin is the one of the VOCs that could explain the vanilla descriptor, since its relative concentration was higher than their OT (Table 1). δ-Cadinol, and acetals, like 1,1-diethoxyethane, *cis*-dioxane, *cis*-dioxolane and *trans*-dioxane could explain the wood descriptor characteristic of older Madeira wines, as their relative concentration slightly increased during aging process (Tables S1 and S2). In regards to the acetals, a little contribution to the sensorial properties of all Madeira wines was expected due to its high OT, and low relative concentration. However, in previous studies, 1,1-diethoxyethane [48] has been considered an important impact odorant to wines and liquors aromas [42,48], despite its higher OT. In the current study, 1,1-diethoxiethane was present in all Madeira wines analyzed at relative concentration lower than its OT.

Dried fruits notes (e.g., almond, coconut, nutty, peanut, walnut) characteristic of old Madeira wines could be explained mainly by the presence of 2-furfural, 5-hydromethyl-2-furfural, (*Z*)-whiskey lactone, γ-octalactone, γ-decalactone, γ-dodecalactone and 1,1-diethoxyethane, as their relative concentration increased remarkably during aging process (Tables S1 and S2). From these VOCs, only (*Z*)-whiskey lactone and γ-decalactone are present at relative concentration higher than its OTs (Table 1).

From a sensorial point of view, as can be observed in Figure 3, during the aging process the wine lost their freshness and fruitness odors related mainly to the presence of terpenic compounds (e.g., linalool oxide, linalool, α-terpeniol, geraniol), norisoprenoids (e.g., TDN, β-damascenone, geranyl acetone), and ethyl esters (e.g., ethyl 3-methylbutanoate, isoamyl acetate, ethyl 3-hydroxyhexanoate), as their relative concentration decreased during aging process (Tables S1 and S2). Otherwise, other descriptors arose such as caramel, dried fruit, spice, toast and wood, that suggests the formation of VOCs by Maillard reaction (e.g., furanic compounds), takes place at 50 °C being favored at pH 4–7 [49], and diffusion from the oak to wines (e.g., lactones, volatile phenols). As can be observed in Figure 3b, the relative concentration of these chemical families increased remarkably during wine aging, which could explain the aroma complexity of older Madeira wines. 

## 3. Materials and Methods 

### 3.1. Sampling

Twenty-two monovarietal Madeira wines from five *Vitis vinifera* L. grape varieties (one red—Tinta Negra, and four white wines from noble varieties—Malvasia, Bual, Sercial, and Verdelho), aged from 3 to 20 years old (Y) and matured in oak casks, were used in this study. Tinta Negra is the main grape variety harvested in Madeira Island (Portugal) representing more than 80% of the vineyards. The grapes were destemmed, crushed and 50 mg/L of sulfur dioxide (SO_2_) was added. According to the age, the wines under study correspond to Vintage (a specific year of aged in casks, 17, 18, 19, and 20 years) and blended wines (B, an average aging period of 3, 5, 10, or 15 years). Four types of wine were used: Sweet (Malvasia, Tinta Negra, sugar content expressed as 96.1 to 150 g glucose per L), medium sweet (Bual, Tinta Negra, 80.4 to 96.1 g/L), dry (Sercial, Tinta Negra, 49.1 to 64.8 g/L), and medium dry (Verdelho, Tinta Negra, 64.8 to 80.4 g/L), and were aged in American oak casks. The ethanol content of the Madeira wines under study ranged from 18 to 19% (*v*/*v*). 

### 3.2. Reagents and Standards

Sodium chloride (99.5%, foodstuff grade) and 4-methyl-2-pentanol (98%, internal standard) was purchased from Sigma Aldrich (Madrid, Spain), and ultrapure water was obtained from a Milli-Q system from Millipore (Milford, MA, USA). α-Pinene, linalool, α-terpeniol, citronellol, geraniol, β-ciclocitral, β-damascenone, geranyl acetone, β-ionone, butan-2-ol, hexan-2-ol, 3-methylbutan-1-ol, hexan-1-ol, (*E*)-3-hexen-1-ol, (*Z*)-3-hexen-1-ol, 2-ethylhexan-1-ol, decan-1-ol, benzyl alcohol, 2-phenethyl alcohol, ethyl butanoate, ethyl 3-methylbutanoate, isoamyl acetate, ethyl hexanoate, hexyl acetate, ethyl lactate, ethyl octanoate, ethyl 3-hydroxybutanoate, ethyl decanoate, ethyl benzeneacetate, ethyl dodecanoate, ethyl salicylate, ethyl 2-phenylacetate, ethyl succinate, acetic acid, butanoic acid, 3-methylbutanoic acid, hexanoic acid, 2-ethylhexanoic acid, octanoic acid, decanoic acid, 2-furfural, 1-(2-furyl)-1-propanone, 5-methyl-2-furfural, ethyl 2-furoate, 5-hydromethyl-2-furfural, butyrolactone, γ-hexalactone, γ-octalactone, γ-decalactone, γ-dodecalactone, 2-phenoxyethanol, eugenol, vanillin and methyl vanillate used for identification of target compounds were purchased from Sigma–Aldrich (Madrid, Spain), Acros Organics (Geel, Belgium), and Fluka (Buchs, Switzerland) with purity higher than 98%. The SPME holder for manual sampling and fiber were purchased from Supelco (Aldrich, Bellefonte, PA, USA). The SPME device included a fused silica fiber coating partially cross-linked with 50/30 μm divinylbenzene/carboxen/polydimethylsiloxane (DVB/CAR/PDMS). Prior to use, the SPME fiber was conditioned at 270 °C for 60 min in the GC injector, according to the manufacturer’s recommendations. Then, the fiber was daily conditioned for 10 min at 250 °C. The *n*-alkane series analytical standard, C_8_ to C_20_ straight-chain alkanes (concentration of 40 mg/L in *n*-hexane), used to determine the Kovat’s index (KI) was supplied from Fluka (Buchs, Switzerland). 

### 3.3. Sensory Analysis

A descriptive sensory analysis of Madeira wines samples used in this study was conducted by a panelist of eleven expert judges (seven females, four males) with an average age of 35 (± 5.1). The eleven members of the panel are winemakers belonging to different wine companies, such Madeira Wines, Barbeito, Borges, Justinos, and some employees from Instituto do Vinho Madeira. The panelists were trained over a period of 70 days to assess wine aroma using a ‘‘Le Nez du Vin” aroma kit (supplied by Ease Scent Company, Beijing, China). The ‘‘Le Nez du Vin” is an aroma kit composed by 54 vials, where each vial contains one typical aroma character in wine, such as blackcurrant, honey, caramel, coffee, chocolate, green pepper, smoke, wood, mint, among others. The training was carried out three times each week for 60–90 min. Each wine (30 mL) was presented to panelists in standard wine tasting glasses coded with three-digit numbers, covered with a Petri dish (to minimize the escape of VOCs), at 19–22 °C, in isolated booths under daylight-type lighting, with randomized presentation order. Cold water was used as palate cleansing. All wines were evaluated in triplicate in three formal sessions that were held on different days.

### 3.4. Headspace Solid-Phase Microextraction Tandem with Gas Chromatography-Mass Spectrometry (HS-SPME) methodology

The HS-SPME experimental parameters were previously established [15]. Briefly, aliquots of 4 mL of the wine sample were placed into an 8 mL glass vial. After the addition of 0.5 g of NaCl, 10 µL of 4-methyl-2-pentanol (internal standard, 250 µg/L) and stirring (0.5 × 0.1 mm bar) at 400 rpm, the vial was capped with a polytetrafluoroethylene (PTFE) septum and an aluminum cap (Chromacol, Hertfordshire, UK). The vial was placed in a thermostatted bath adjusted to 40.0 ± 0.1 °C for 5 min, and then the DVB/CAR/PDMS fiber was inserted into the headspace for 30 min. Three independent aliquots of each sample were analyzed in triplicate. Blanks, corresponding to the analysis of the coating fiber not submitted to any extraction procedure, were run between sets of three analysis.

### 3.5. GC–qMS Analysis for Madeira Wines Profiling

The GC–qMS methodology was based on a previous study [50]. After the extraction/concentration step, the SPME coating fiber containing the VOCs was manually introduced into the GC injection port at 250 °C (equipped with a glass liner, 0.75 mm I.D.) and kept for 7 min for desorption. The desorbed VOCs were separated in an Agilent Technologies 6890N Network gas chromatography equipped with a BP-20 fused silica capillary column (30 m × 0.25 mm I.D. × 0.25 μm film thickness) supplied by SGE (Darmstadt, Germany) connected to an Agilent 5973N quadrupole mass selective detector. Helium (Air Liquid, Portugal) was used as the carrier gas at a flow rate of 1.0 mL/min (column-head pressure: 12 psi). The injections were performed in the splitless mode (7 min). The GC oven temperature was programmed as follows: 45 °C (1 min) then ramped at 2 °C/min to 100 °C (3 min), 5 °C/min to 130 °C (5 min), and finally 2 °C/min to 220 °C (2 min). For the MS system, the temperatures of the transfer line, quadrupole and ionization source were 250, 150 and 230 °C, respectively; electron impact mass spectra were recorded at 70 eV and the ionization current was about 30 μA. The acquisitions were performed in full scan mode (30–300 *m/z*). The VOCs identification was achieved as follows: (*i*) comparison the GC retention times and mass spectra with those of the standard, when available; (*ii*) all mass spectra were also compared with the data system library (NIST, 2005 software, Mass Spectral Search Program v.2.0d; Nist 2005, Washington, DC); (*iii*) Kovat’s index (KI) values were determined according to the van den Dool and Kratz equation [51]. For the KI determination, a C_8_–C_20_ n-alkanes series was used, and the values were compared, when available, with values reported in the literature for similar columns [21,22,23,24,25,26,27,28,29,30].

The VOCs concentration was estimated, semi quantitatively, using the added amount of 4-methyl-2-pentanol (IS) according the following equation: VOCs concentration = (VOC GC peak area/IS GC peak area) × IS concentration. However, our main aim is regarding the relation between the varietal, fermentative and aging aroma compounds, and their aroma descriptors with the Madeira wine sensorial properties. This semi quantification approach was already performed in previous scientific studies [52,53].

## 4. Conclusions

This study represents the first detailed research about the Madeira wines volatile profiling and its association with odor descriptors. An in-depth relation among the varietal, fermentative and aging aroma compounds and their odor descriptors with the Madeira wine sensory analysis (described by a trained panelist) was established. The Madeira wine aroma notes, the VOCs and their aroma descriptors, showed the data complexity and the difficulty to get information. From the aroma system, it can be verified that grape variety is an important parameter that influences the sensorial properties of young Madeira wines, whereas the old wines are highly influenced by the aging process. 

From a sensorial point of view, during the aging process the wine lost its freshness and fruitiness odors, and other descriptors arise such as caramel, dried fruit, spice, toast and wood, that suggests the formation of VOCs by Maillard reaction (e.g., furanic compounds), and diffusion from the oak to wines (e.g., lactones, volatile phenols). In addition, young Madeira wines obtained from Malvasia and Bual grape varieties are more complex than those obtained from Tinta Negra, Sercial, and Verdelho. This trend is not observed for the old Madeira wines since, independently of the grape variety used, their aroma notes are balanced which means that aging process has a higher impact on aroma rather than grape variety.

It is important to point out that a detailed database about volatile composition of Madeira wine and the correspondent aroma descriptors was built, which may be useful to improve information about the specific aroma of Madeira wine and will represent a powerful tool to help on winemaker decisions.

## Figures and Tables

**Table 1:** Volatile organic compounds (VOCs) identified in Madeira wines by headspace solid-phase microextraction tandem with gas chromatography-mass spectrometry (HS–SPME/GC–qMS), and the corresponding odor thresholds and odor descriptors.

RT (min) ^1^	KI_calc_^2^	KI_lit_^3^	ID ^4^	Chemical Families	OTs (µg/L) ^5^	Odor descriptors ^6^
				**Terpenic compounds**		
7.34	1150	1158	MS, RI, Std	β-Pinene	6	**^7^ Citrus**, **floral**, fruit, green, pine, sweet, terpenic, **wood**
8.38	1178	1182	MS, RI, Std	**Limonene ^8^**	10	**Citrus**, fruit, **wood**
18.96	1430	1433	MS, RI	**(*E*)-Linalool oxide**	500	**Floral**, green, rose, sweet
19.16	1445	1451	MS, RI	**(*Z*)-Linalool oxide**	500	**Floral**, green, rose, sweet
22.88	1537	1537	MS, RI, Std	**Linalool**	15	**Citrus**, lavender, **floral**, fruit, green, muscat, sweet
24.01	1561	1566	MS, RI, Std	**β-Caryophyllene**	- ^9^	Fruit, green, **spice**, **wood**
28.58	1673	1669	MS, RI, Std	**α-Terpineol**	250	Anise, **floral**, fruit, mint, oil, toothpaste
31.38	1764	1762	MS, RI, Std	Citronellol	30	**Citrus**, clove, **floral**, fresh, green, rose, sour, sweet
39.86	1981	2009	MS, RI, Std	**Geraniol**	20	**Citrus**, **floral**, fruit, **waxy**
41.99	2125	2134	MS, RI	δ-Cadinol	-	**Almond**, green, **waxy**, **wood**
				**Norisoprenoids**		
21.47	1498	1507	MS, RI, Std	**Vitispirane I**	800	Camphor, eucalyptus, **spice**, **wood**
21.57	1501	1510	MS, RI, Std	**Vitispirane II**	-	Camphor, eucalyptus, **spice**, **wood**
26.22	1614	1623	MS, RI, Std	**β-Cyclocitral**	5	**Floral**, sweet
30.86	1742	1755	MS, RI, Std	**TDN ^10^**	2	**Floral**, fruit, pleasant, wine
32.33	1785	1790	MS, RI, Std	**β-Damascenone ^6^**	0.05	**Floral**, fruit, **honey**, sweet, **tobacco**
34.79	1844	1840	MS, RI, Std	**Geranyl acetone**	60	**Floral**, fruit, green, **waxy**, **wood**
36.34	1910	1912	MS, RI, Std	β-Ionone	0.10	**Floral**, violet
				**Higher Alcohols**		
4.78	1074	1057	MS, RI, Std	**Butan-2-ol**	-	Alcohol, oil, wine
6.12	1113	1112	MS, RI, Std	**2-Methylpropan-1-ol**	40,000	Alcohol, bitter, glue, leek, licorice
7.87	1165	1176	MS, RI, Std	**Hexan-2-ol**	-	Fatty, fruit, wine
9.51	1206	1206	MS, RI, Std	**3-Methylbutan-1-ol**	30,000	Alcohol, balsamic, burnt, cheesy, fruit, pungent, ripe onion
15.15	1350	1354	MS, RI, Std	**Hexan-1-ol**	8000	**Floral**, fruit, green, herbal, mild, **toasty**, sweet, **wood**
15.23	1352	1362	MS, RI, Std	**(*E*)-3-Hexen-1-ol**	400	Fresh, green, grass, leaf
16.11	1371	1379	MS, RI, Std	**(*Z*)-3-Hexen-1-ol**	400	Fresh, green, grass, leaf
22.25	1514	1510	MS, RI, Std	**2-Ethylhexan-1-ol**	270	**Citrus**, fresh, **floral**, oil, sweet
22.43	1521	1524	MS, RI	**(*R*,*S*)-Butan 2,3-diol**	120,000	Fruit
23.93	1556	1556	MS, RI	**(*R*,*R*)-Butan-2,3-diol**	120,000	Fruit
32.29	1784	1783	MS, RI, Std	**Decan-1-ol**	400	Fatty
35.31	1861	1869	MS, RI, Std	**Benzyl alcohol**	200,000	Blackberry, **floral**, fruit
36.42	1915	1910	MS, RI, Std	**2-Phenyethyl alcohol**	14,000	**Floral**, herbal, **honey**, pollen, rose, **spice**, sweet
				**Sulphur compound**		
29.65	1701	1723	MS, RI, Std	Ethyl 3-(methylthio)propionate	7	Fruit, pineapple
				**Esters**		
4.68	1055	1047	MS, RI, Std	**Ethyl butanoate**	20	Acetone, bubblegum, **caramel**, fruit
4.71	1061	1053	MS, RI, Std	**Ethyl 3-methylbutanoate**	3	Anise, apple, blackcurrant, **citrus**, fruit, sweet
6.51	1125	1120	MS, RI, Std	**Isoamyl acetate**	30	**Banana**, fresh, fruit, sweet
10.04	1222	1220	MS, RI, Std	**Ethyl hexanoate**	5	Anise, **caramel**, fruit, wine
10.31	1254	1262	MS, RI, Std	**Hexyl acetate**	10	Acid, **citrus**, fruit, green, herbal, rubber, **spice**, **tobacco**
10.52	1279	1276	MS, RI, Std	**Ethyl pyruvate**	-	**Caramel**, **ethereal**, fruit, vegetable, sweet
14.67	1339	1340	MS, RI	**Ethyl lactate**	154,636	Acidic, **ethereal**, fruit, sweet
18.16	1416	1414	MS, RI, Std	**Ethyl octanoate**	2	Fruit, must, soap, sweet, **waxy**
20.98	1478	1483	MS, RI, Std	**Ethyl 3-hydroxybutanoate**	20,000	Coconut, grape, **nutty**
26.58	1617	1624	MS, RI, Std	**Ethyl decanoate**	200	Fruit, pleasant, soap, sweet, **waxy**
28.07	1659	1661	MS, RI, Std	**Diethyl succinate**	500,000	Fabric, floral, fruit, lavender, potato, sweat
29.34	1693	1696	MS, RI	**Ethyl 3-hydroxyhexanoate**	265	**Citrus**, fruit, green, sweet
29.72	1715	1708	MS, RI	**Ethyl 9-decenoate**	100	Fruit, fatty
32.07	1773	1775	MS, RI, Std	**Ethyl benzeneacetate**	-	Fruit
34.52	1838	1837	MS, RI, Std	**Ethyl dodecanoate**	500	Fruit, soap, sweet
34.99	1857	1839	MS, RI, Std	**Ethyl salicylate**	-	Balsamic, cooling, **floral**, fruit, **spice**, sweet
35.58	1873	1870	MS, RI	**Benzyl butanoate**	-	**Floral**, fruit, jasmin, sweet,
35.87	1880	1883	MS, RI, Std	**Ethyl 2-phenylacetate**	250	**Floral**
66.75	2354	2358	MS, RI, Std	**Diethyl tartrate**	-	-
68.62	2420	2440	MS, RI, Std	**Ethyl succinate**	-	Fruit
70.49	2486	2499	MS, RI	**Ethyl citrate**	-	**Floral**
				**Acids**		
18.51	1425	1426	MS, RI, Std	**Acetic acid**	200,000	Pungent, vinegar, sour
23.52	1547	1557	MS, RI, Std	**2** **-Methylpropanoic acid**	200,000	Cheesy, fatty, phenolic, sweaty
25.97	1600	1607	MS, RI, Std	**Butanoic acid**	10,000	Buttery, cheesy, rancid, sweaty
27.56	1645	1647	MS, RI, Std	**3-Methylbutanoic acid**	3000	Cheesy, rancid, sweaty
34.49	1837	1840	MS, RI, Std	**Hexanoic acid**	3000	Cheesy, pungent, rancid, sweaty
36.92	1978	1981	MS, RI, Std	**2-Ethylhexanoic acid**	-	Cheesy
41.82	2098	2089	MS, RI, Std	**Octanoic acid**	10,000	Cheesy, fatty, fresh, moss
48.56	2321	2317	MS, RI, Std	**Decanoic acid**	15,000	Cheesy, fatty, soap
67.81	2392	2407	MS, RI	**Undecylic acid**	40	Oil
				**Acetals**		
4.99	1094	1096	MS, RI, Std	**1,1-Diethoxyethane**	1000	Liquorices, **nutty**, pungent, **wood**
21.86	1512	1525	MS, RI, Std	***Cis*-dioxane**	-	**Wood**
26.84	1642	1639	MS, RI, Std	***Cis*-dioxolane**	-	**Wood**
31.01	1755	1740	MS, RI, Std	***Trans*-dioxane**	-	**Wood**
				**Furanic compounds**		
18.03	1412	1434	MS, RI, Std	**2-Acetylfuran**	-	Balsamic-cinnamic, cereal, sweet, **toast**, **tobacco**
20.86	1465	1458	MS, RI, Std	**2-Furfural**	14,100	**Almond**, **caramel**, sweet, **wood**
22.45	1526	1524	MS, RI, Std	**1-(2-Furyl)-1-propanone**	-	Radish, **spice**
23.67	1550	1560	MS, RI, Std	**5-Methyl-2-furfural**	20,000	Acid, **almond**, **caramel**, coffee, **spice**, **toast**
26.01	1606	1606	MS, RI, Std	**Ethyl 2-furoate**	16,000	Balsamic, scorched tone, **vanilla**
68.15	2412	-	MS, RI, Std	**5** **-Ethoxymethyl-2-furfural**	6	Curry, **spice**
75.02	2501	2509	MS, RI, Std	**5-Hydroxymethyl-2-furfural**	10,000	**Almond**, cardboard, **nutty**
				**Lactones**		
25.71	1594	1595	MS, RI, Std	Butyrolactone	35,000	**Caramel**, coconut, cream, peach
29.01	1690	1694	MS, RI, Std	γ-Hexalactone	1600	Apricot, peach
36.65	1936	1933	MS, RI, Std	γ-Octalactone	400	**Caramel**, coconut, cream, fatty, herbaceous, **nutty**
42.75	2197	2185	MS, RI, Std	γ-Decalactone	88	Fruit, sweet
43.92	2218	2219	MS, RI	(Z)-Whiskylactone	67	**Caramel**, coconut, **nutty**, **toast**, **wood**
45.44	2267	2241	MS, RI, Std	γ-Dodecalactone	1000	Coconut, fruit, musk, sweet
				**Volatile phenols**		
41.63	2076	2080	MS, RI, Std	**2-Phenoxyethanol**	-	Alcoholic, **floral**, rose
45.13	2257	2250	MS, RI, Std	**Eugenol**	5	Balsamic, clove, herbaceous, **honey**, **spice**
77.06	2563	2561	MS, RI, Std	Vanillin	4	Sweet, **vanilla**
78.77	2620	2613	MS, RI, Std	Methyl vanillate	990	**Vanilla**

^1^ Retention time (min); ^2^ Kovats index *n*-alkanes (C_8_ to C_20_) on a BP-20 capillary column; ^3^ Kovats index reported in literature for equivalent capillary column [21,22,23,24,25,26,27,28,29,30]; ^4^ Method of identification: MS, mass spectrum comparison using NITS library; RI: retention index in agreement with literature value; Std: confirmed by authentic standard; ^5^ Odor threshold determined in 10–12% *v/v* ethanol [6,31,32,33,34,35,36]; ^6^ Odor descriptors [6,11,37,38,39,40,41,42,43,44,45]; ^7^ Odor descriptors in bold are the potential aroma notes of Madeira wines; ^8^ VOCs in bold are common to all Madeira wines analyzed; ^9^ No information was found in literature; ^10^ TDN: 1,2-dihydro-1,1,6-trimethylnaphtalene.

**Table 2:** Madeira wines sensory analysis obtained from different grape varieties, types, and ages.

Madeira Wine Sensory Analysis		
Variety	Younger wines (3 to 5 Years Old)	Older wines (10 to 20 Years Old)
Malvasia	Almond, banana, citrus, cocoa, floral, tobacco, wood	Almond, caramel, dried fruits, spice, tobacco, toast, vanilla, wood
Bual	Almond, banana, cocoa, floral, tea	Almond, caramel, dried fruits, spice, tea, toast, wood
Sercial	Citrus, honey, mushroom, waxy	Dried fruits, honey, spice, toast, vanilla, wood
Verdelho	Banana, floral, honey, mushroom, spice	Dried fruits, ethereal, honey, spice, toast, wood
Tinta Negra	Citrus, ripe fruit, tea, wood	Caramel, dried fruits, spice, tea, toast, wood

**Table 3:** Potential impact odorants of Madeira wine.

Odor Descriptor	Madeira Wines	Potential Odorant
Citrus	Malvasia, Sercial, TN	α-pinene, limonene, linalool, citronellol, geraniol, hexyl acetate, ethyl 3-methylbutanoate, ethyl 3-hydroxyhexanoate, 2-ethylhexan-1-ol
Floral	Malvasia, Bual, Verdelho	α-pinene, linalool, citronellol, geraniol, β-cyclocitral, TDN ^1^, β-damascenone, geranyl acetone, β-ionone, 1-hexanol, 2-phenylethyl alcohol
Waxy	Sercial	geraniol, geranyl acetone, ethyl octanoate, ethyl decanoate
Almond	Malvasia, Bual, Tinta Negra	δ-cadinol
Caramel	Malvasia, Bual	ethyl butanoate, ethyl hexanoate, ethyl pyruvate, (*Z*)-whiskylactone
Ethereal	Verdelho	ethyl lactate, ethyl pyruvate
Spice	Malvasia, Bual, Verdelho, Sercial, TN	hexyl acetate, 2-phenyethyl alcohol, 5-(ethoxymethyl)furfural, eugenol
Toast	Malvasia, Bual, Verdelho, Sercial, TN	(*Z*)-whiskylactone
Wood	Malvasia, Bual, Verdelho, Sercial, TN	δ-cadinol
Vanilla	Malvasia, Sercial	ethyl 2-furoate, vanillin, methyl vanillate

^1^ TDN: 1,2-dihydro-1,1,6-trimethylnaphthalene; TN: Tinta Negra.

## Supplementary Materials

The following are available online, Table S1: Relative concentration (µg/L) and relative standard deviation (%RSD) of VOCs identified by HS-SPME/GC-qMS in dry (Sercial and Tinta Negra) and medium dry (Verdelho and Tinta Negra) Madeira wines, Table S2: Relative concentration (µg/L) and relative standard deviation (RSD) of VOCs identified by HS-SPME/GC-qMS in sweet (Malvasia and Tinta Negra) and medium sweet (Bual and Tinta Negra) Madeira wines.
