# Supplementary material for: Madeira Wine Volatile Profile. A Platform to Establish Madeira Wine Aroma Descriptors
Source: Molecules. 2019 Aug 21;24(17):3028. doi: 10.3390/molecules24173028 (PMC6749321; doi:10.3390/molecules24173028)
Supplement: Supplementary file 1 [file molecules-24-03028-s001.pdf]

Supplementary materials

Madeira Wine volatilome. A platform to establish Madeira wine aroma descriptors

Rosa Perestrelo[a], Catarina Silva[a], José S. Câmara[a,b]

[a] *CQM – Centro de Química da Madeira, Universidade da Madeira, Campus da Penteada, 9020-105 Funchal, Portugal*
[b] *Departamento de Química, Faculdade de Ciências Exatas e Engenharia, Universidade da Madeira, Campus da Penteada, 9020-105 Funchal, Portugal*

Table S1: Relative concentration (μg/L) and relative standard deviation (%RSD) of VOCs identified by HS-SPME/GC-qMS in dry (Sercial and Tinta Negra) and medium dry (Verdelho and Tinta Negra) Madeira wines.

Chemical families	Dry								Medium dry							
	Sercial				Tinta Negra				Verdelho				Tinta Negra			
	5Y B[1]		10Y B		3Y B		5Y B		5Y B		10Y[2]		3Y B		5Y B	
**Terpenic compounds**																
α-Pinene	9.09	(16)	8.14	(7)	9.14	(12)	8.61	(17)	9.18	(2)	-	-	9.18	(13)	8.35	(9)
Limonene	18.88	(2)	18.70	(10)	18.85	(6)	18.67	(10)	19.39	(18)	18.78	(16)	18.98	(7)	18.54	(4)
(*E*)-Linalool oxide	0.67	(6)	0.61	(6)	1.28	(3)	0.81	(4)	1.31	(8)	0.87	(18)	0.57	(18)	0.69	(16)
(*Z*)-Linalool oxide	2.17	(6)	1.92	(4)	2.10	(10)	3.01	(7)	2.21	(11)	3.26	(18)	1.30	(16)	0.67	(11)
Linalool	95.15	(5)	92.17	(4)	140.93	(8)	136.47	(1)	140.50	(12)	138.34	(2)	142.33	(18)	138.53	(12)
β-Caryophyllene	5.45	(13)	5.21	(18)	5.27	(11)	2.51	(15)	5.16	(4)	0.19	(4)	4.77	(7)	6.73	(4)
α-Terpineol	81.48	(5)	65.39	(2)	79.45	(4)	84.92	(11)	91.11	(2)	53.98	(17)	121.21	(6)	66.78	(12)
Citronellol	-	-	-	-	40.72	(2)	36.48	(12)	40.86	(16)	-	-	40.66	(12)	36.62	(8)
Geraniol	63.00	(4)	56.10	(10)	64.50	(13)	55.23	(10)	64.67	(8)	46.04	(18)	64.49	(4)	55.18	(11)
δ-Cadinol	0.50	(14)	-	-	0.72	(4)	-	-	0.56	(16)	0.22	(7)	0.70	(5)	1.00	(5)
**Norisoprenoids**																
Vitispirane I	29.95	(3)	26.85	(9)	23.52	(7)	22.56	(7)	28.08	(7)	16.59	(10)	27.86	(6)	30.60	(3)
Vitispirane II	2.94	(1)	2.34	(6)	2.58	(1)	3.51	(8)	2.54	(5)	1.92	(11)	2.61	(8)	3.94	(7)
β-Cyclocitral	18.07	(2)	15.93	(1)	18.28	(5)	16.52	(1)	18.19	(6)	16.68	(15)	18.37	(12)	16.36	(16)
TDN	18.91	(1)	21.99	(2)	22.22	(3)	7.22	(3)	22.61	(1)	3.10	(9)	18.69	(4)	18.15	(1)
β-Damascenone	106.57	(3)	101.67	(8)	89.22	(1)	87.03	(8)	89.39	(3)	73.46	(8)	94.50	(7)	88.56	(11)
Geranyl acetone	147.35	(1)	133.98	(1)	138.60	(3)	126.92	(13)	144.64	(1)	135.91	(1)	142.28	(1)	140.01	(18)
β-Ionone	-	-	0.79	(1)	0.18	(6)	-	-	0.66	(1)	0.69	(11)	0.29	(2)	0.99	(12)
**Alcohols**																
Butan-2-ol	4.74	(4)	1.96	(10)	0.75	(1)	2.26	(2)	1.88	(4)	4.07	(11)	2.42	(18)	4.30	(9)
2-Methylpropan-1-ol	0.70	(6)	0.57	(5)	0.76	(8)	0.35	(9)	0.43	(1)	0.36	(9)	0.67	(12)	0.60	(14)
Hexan-2-ol	1.36	(2)	1.37	(17)	0.50	(1)	0.84	(4)	1.75	(8)	0.86	(8)	0.54	(8)	1.36	(12)
3-Methylbutan-1-ol	183414.79	(1)	205118.42	(7)	190760.18	(1)	152172.52	(5)	219028.27	(1)	224412.40	(6)	174607.21	(4)	199734.33	(6)
Hexan-1-ol	164.40	(7)	252.61	(3)	224.19	(3)	283.38	(12)	108.35	(18)	184.66	(2)	126.00	(6)	133.44	(7)
(*E*)-3-Hexen-1-ol	89.23	(5)	78.40	(25)	145.65	(9)	163.20	(1)	138.19	(8)	141.34	(9)	83.02	(16)	98.99	(9)
(*Z*)-3-Hexen-1-ol	98.17	(2)	101.62	(4)	89.82	(1)	96.45	(8)	67.96	(13)	47.75	(10)	94.14	(2)	108.28	(1)
2-Ethylhexan-1-ol	419.24	(7)	329.36	(15)	457.68	(9)	408.72	(8)	373.35	(5)	366.48	(12)	413.06	(15)	323.04	(5)
(*R*,*S*)-Butan 2,3-diol	86.41	(17)	58.46	(15)	83.78	(9)	66.61	(9)	93.22	(18)	59.34	(18)	75.26	(3)	89.47	(4)
(*R*,*R*)-Butan-2,3-diol	18.96	(3)	18.17	(3)	35.02	(5)	24.79	(15)	18.76	(7)	15.22	(3)	32.44	(10)	26.50	(11)
Decan-1-ol	4.46	(6)	5.21	(6)	3.58	(1)	3.13	(16)	4.94	(7)	0.64	(15)	4.08	(1)	4.83	(1)
Benzyl alcohol	4224.04	(5)	4315.42	(8)	2654.97	(4)	4135.95	(13)	3821.88	(2)	2565.58	(16)	2475.73	(8)	5123.14	(7)
2-Phenyethyl alcohol	23816.09	(1)	22622.15	(5)	20827.13	(1)	21993.90	(4)	17507.10	(9)	13289.29	(8)	21949.04	(1)	26121.86	(8)
**Sulphur components**																
Ethyl 3-(methylthio)propionate	0.85	(9)	0.50	(6)	2.60	(10)	3.14	(10)	1.02	(6)	0.56	(11)	1.79	(11)	2.26	(14)
**Esters**																
Ethyl butanoate	96.00	(13)	146.21	(3)	56.18	(7)	56.43	(11)	146.17	(11)	280.48	(7)	190.97	(19)	235.73	(10)
Ethyl 3-methylbutanoate	10.20	(1)	9.07	(6)	6.78	(19)	4.03	(2)	19.50	(3)	9.20	(18)	20.35	(12)	2.67	(1)
Isoamyl acetate	467.91	(9)	349.94	(1)	372.30	(11)	331.90	(7)	412.61	(7)	381.12	(5)	246.69	(8)	214.93	(6)
Ethyl hexanoate	9294.23	(18)	7095.29	(4)	5480.31	(14)	4403.11	(3)	9383.95	(4)	7454.49	(4)	6153.19	(5)	5794.26	(5)
Hexyl acetate	83.95	(2)	82.92	(3)	83.11	(4)	78.74	(6)	82.99	(1)	74.47	(15)	82.69	(13)	73.54	(6)
Ethyl pyruvate	2.91	(10)	2.27	(2)	0.85	(12)	1.51	(9)	3.09	(3)	3.98	(12)	0.68	(4)	1.06	(4)
Ethyl lactate	16325.97	(5)	12511.71	(4)	10223.13	(9)	10851.48	(8)	13768.16	(5)	8024.66	(6)	12870.38	(2)	16370.35	(2)
Ethyl octanoate	1117.15	(6)	977.57	(16)	1067.93	(21)	717.13	(12)	986.53	(11)	665.09	(17)	1314.08	(11)	709.47	(7)
Ethyl 3-hydroxybutanoate	175.79	(2)	186.58	(3)	189.22	(4)	144.15	(9)	188.96	(2)	189.32	(8)	99.40	(18)	96.77	(9)
Ethyl decanoate	8920.32	(1)	7274.04	(20)	10912.77	(6)	6825.82	(7)	5169.83	(11)	3823.62	(1)	5887.66	(3)	4048.02	(8)
Diethyl succinate	22357.32	(5)	27426.16	(1)	30118.24	(2)	56591.50	(15)	103704.19	(2)	112229.62	(17)	109088.81	(1)	114921.76	(11)
Ethyl 3-hydroxyhexanoate	0.31	(10)	0.49	(11)	0.19	(5)	0.37	(25)	0.33	(7)	0.51	(21)	0.29	(5)	0.34	(8)
Ethyl 9-decenoate	27.73	(6)	15.80	(3)	14.07	(5)	28.77	(7)	15.09	(1)	14.98	(9)	8.27	(10)	10.44	(9)
Ethyl benzeneacetate	1.99	(9)	1.84	(6)	1.05	(2)	1.33	(10)	1.91	(3)	1.63	(12)	1.20	(10)	1.57	(9)

Ethyl dodecanoate	391.21	(9)	590.53	(2)	815.26	(7)	520.72	(14)	635.76	(3)	724.53	(18)	590.75	(8)	770.31	(9)
Ethyl salicylate	0.38	(14)	3.79	(13)	0.17	(5)	0.30	(11)	0.89	(13)	0.85	(13)	0.27	(13)	0.41	(6)
Benzyl butanoate	12.73	(6)	13.51	(12)	12.29	(12)	13.99	(9)	12.80	(1)	8.48	(10)	8.89	(8)	13.03	(3)
Ethyl 2-phenylacetate	2875.50	(5)	3911.24	(8)	1981.61	(7)	2116.13	(4)	3821.53	(1)	3821.40	(6)	2654.70	(2)	2834.24	(4)
Diethyl tartarate	1.06	(17)	1.71	(6)	0.52	(5)	0.56	(1)	1.46	(11)	1.06	(14)	0.97	(16)	0.85	(14)
Ethyl succinate	94.43	(27)	142.03	(5)	52.46	(4)	52.17	(15)	187.07	(9)	97.21	(3)	97.11	(1)	142.02	(7)
Ethyl citrate	0.38	(14)	3.79	(13)	0.17	(5)	0.30	(11)	0.89	(13)	0.85	(13)	0.27	(13)	0.41	(6)
**Acids**																
Acetic acid	7946.39	(1)	6065.68	(1)	4584.77	(2)	6648.88	(1)	4046.36	(2)	4046.69	(1)	5796.66	(7)	5796.28	(4)
2-Methylpropanoic acid	0.45	(5)	0.51	(2)	0.45	(17)	0.61	(2)	1.02	(4)	0.89	(3)	0.93	(3)	0.89	(7)
Butanoic acid	1.83	(9)	1.92	(7)	2.06	(9)	2.37	(4)	1.62	(5)	1.82	(2)	1.73	(4)	1.59	(6)
3-Methylbutanoic acid	94.33	(2)	94.29	(17)	94.45	(5)	94.51	(4)	4.64	(14)	4.66	(16)	4.84	(5)	4.85	(3)
Hexanoic acid	6340.02	(3)	6115.39	(14)	4051.65	(7)	3199.14	(10)	5666.99	(4)	7327.09	(3)	4634.63	(9)	7237.25	(7)
2-Ethylhexanoic acid	2.30	(3)	2.14	(5)	0.80	(9)	0.55	(9)	1.91	(8)	2.23	(5)	0.92	(10)	1.02	(2)
Octanoic acid	5061.80	(1)	4616.65	(2)	4168.23	(3)	4168.23	(7)	2615.53	(2)	2615.47	(5)	3512.90	(4)	3513.26	(2)
Decanoic acid	3689.01	(8)	3240.09	(8)	2791.53	(2)	2791.49	(14)	2791.56	(10)	2343.01	(4)	2791.33	(2)	2342.98	(8)
Undecylic acid	0.39	(14)	0.42	(15)	0.44	(19)	0.46	(9)	0.49	(8)	0.47	(3)	0.66	(3)	0.67	(2)
**Acetals**																
1,1-Diethoxyethane	60.07	(4)	69.02	(6)	67.97	(4)	72.50	(2)	68.70	(3)	83.36	(2)	94.95	(5)	112.75	(4)
Cis-dioxane	116.21	(6)	159.60	(1)	98.52	(8)	143.64	(2)	110.63	(4)	130.60	(7)	141.50	(2)	172.40	(2)
Cis-dioxolane	44.82	(5)	52.08	(7)	42.06	(3)	49.05	(3)	47.01	(6)	80.86	(4)	51.25	(3)	62.61	(3)
Trans-dioxane	-	-	41.55	(4)	-	-	43.25	(5)	43.24	(2)	49.94	(3)	41.86	(7)	47.40	(10)
**Furanic compounds**																
2-Acetylfuran	6.49	(5)	8.36	(7)	4.52	(5)	6.68	(6)	7.92	(2)	18.88	(1)	5.50	(8)	8.39	(3)
2-Furfural	84.52	(2)	102.33	(4)	107.02	(8)	142.94	(3)	120.52	(1)	133.75	(2)	111.59	(2)	125.05	(1)
1-(2-Furyl)-1-propanone	1.19	(10)	1.08	(2)	0.48	(4)	0.48	(9)	1.15	(8)	2.45	(11)	0.78	(10)	1.35	(3)
5-Methyl-2-furfural	26.28	(8)	26.16	(5)	9.68	(3)	12.66	(4)	22.87	(3)	37.30	(4)	15.37	(8)	26.18	(2)
Ethyl 2-furoate	4.16	(11)	13.34	(6)	17.76	(8)	22.05	(8)	30.97	(2)	40.05	(6)	22.30	(5)	31.13	(4)
5-Ethoxymethyl-2-furfural	5.36	(14)	9.48	(3)	0.92	(9)	1.02	(5)	6.63	(4)	12.78	(9)	2.67	(3)	3.72	(5)
5-Hydroxymethyl-2-furfural	7.17	(7)	35.57	(9)	35.02	(10)	49.07	(6)	49.34	(2)	71.58	(6)	26.81	(2)	53.42	(3)
**Lactones**																
Butyrolactone	1.14	(5)	2.61	(1)	0.60	(2)	1.95	(14)	-	-	1.76	(3)	0.54	(14)	1.04	(2)
γ-Hexalactone	153.13	(2)	154.33	(6)	152.75	(1)	154.27	(9)	152.90	(15)	154.69	(7)	-	-	158.24	(5)
γ-Octalactone	-	-	229.17	(4)	184.18	(8)	193.14	(17)	-	-	202.18	(11)	206.70	(17)	201.02	(7)
γ-Decalactone	157.35	(12)	166.29	(3)	-	-	166.30	(11)	-	-	170.61	(18)	179.81	(8)	184.49	(3)
(*Z*)-Whiskylactone	63.59	(8)	69.03	(5)	94.49	(19)	99.31	(7)	99.52	(1)	109.80	(1)	-	-	113.08	(10)
γ-Dodecalactone	-	-	0.11	(11)	-	-	0.07	(1)	-	-	0.06	(9)	0.13	(20)	0.38	(8)
**Volatile phenols**																
2-Phenoxyethanol	0.82	(12)	0.26	(18)	0.14	(19)	0.23	(5)	0.88	(2)	0.12	(18)	0.35	(14)	1.70	(3)
Eugenol	5.08	(6)	7.26	(5)	9.49	(8)	13.77	(7)	9.51	(3)	12.08	(18)	9.88	(17)	11.85	(10)
Vanillin	-	-	18.77	(8)	-	-	-	-	-	-	22.70	(15)	22.66	(9)	28.15	(2)
Methyl vanillate	0.34	(18)	0.66	(1)	-	-	0.46	(16)	0.45	(13)	2.53	(5)	0.25	(18)	0.54	(1)

[1] YB - blend wine - years, average age; [2] Y - vintage wines - age expressed in years; - : not detected; TDN: 1,2-Dihydro-1,1,6-trimethylnaphthalene

Table S2: Relative concentration (µg/L) and relative standard deviation (%RSD) of VOCs identified by HS-SPME/GC-qMS in sweet (Malvasia and Tinta Negra) and medium sweet (Bual and Tinta Negra) Madeira wines.

Chemical families	Sweet							Medium sweet						
	Malvasia					Tinta Negra		Bual					Tinta Negra	
	5Y B[1]	10Y B	15Y B	18Y[2]	20Y	3Y B	5Y B	5Y B	10Y B	15Y B	17 Y	19 Y	3Y B	5Y B
**Terpenic compounds**														
α-Pinene	9.18 (15)	9.04 (8)	9.07 (20)	9.06 (33)	9.05 (7)	9.06 (14)	9.10 (3)	9.27 (20)	9.17 (3)	9.24 (9)	9.18 (8)	9.08 (8)	9.10 (15)	9.19 (20)
Limonene	28.14 (17)	28.25 (1)	28.21 (12)	29.49 (20)	28.32 (6)	27.88 (1)	28.42 (2)	27.68 (3)	27.69 (3)	28.11 (11)	28.12 (6)	27.60 (18)	27.77 (7)	28.03 (5)
(*E*)-Linalool oxide	5.24 (14)	4.34 (7)	7.31 (14)	7.86 (7)	8.68 (10)	3.06 (3)	4.16 (8)	4.75 (15)	3.59 (8)	8.33 (16)	5.47 (11)	4.75 (19)	3.73 (9)	3.52 (11)
(*Z*)-Linalool oxide	2.21 (7)	2.59 (8)	2.50 (3)	3.71 (16)	4.05 (10)	0.95 (7)	1.27 (9)	1.62 (6)	1.15 (18)	3.68 (9)	3.56 (8)	2.36 (12)	0.89 (7)	1.37 (5)
Linalool	144.42 (9)	144.30 (10)	144.22 (10)	144.37 (5)	144.08 (2)	139.98 (9)	139.88 (12)	139.99 (10)	139.81 (7)	139.58 (18)	139.58 (8)	102.61 (15)	139.60 (19)	102.43 (11)
β-Caryophyllene	10.09 (3)	8.78 (11)	1.72 (4)	1.97 (5)	1.61 (8)	5.59 (5)	1.58 (7)	3.13 (5)	0.56 (9)	4.79 (8)	2.01 (5)	2.71 (13)	7.67 (15)	5.70 (3)
α-Terpineol	94.35 (10)	56.36 (1)	70.91 (8)	51.63 (9)	47.27 (3)	110.01 (4)	83.14 (2)	110.08 (6)	51.77 (14)	56.44 (7)	42.93 (3)	51.81 (12)	78.73 (5)	75.49 (5)
Citronellol	45.48 (9)	49.72 (6)	- -	- -	- -	49.50 (2)	- -	49.46 (3)	49.43 (7)	- -	- -	- -	49.55 (17)	- -
Geraniol	73.77 (9)	73.73 (8)	73.67 (13)	73.92 (5)	73.62 (4)	74.10 (6)	73.82 (9)	73.78 (4)	73.16 (11)	73.46 (5)	73.01 (13)	73.32 (15)	72.89 (8)	73.20 (12)
δ-Cadinol	0.92 (19)	0.49 (11)	- -	- -	- -	- -	- -	0.94 (5)	0.19 (18)	- -	- -	- -	0.91 (21)	0.94 (15)
**Norisoprenoids**														
Vitispirane I	19.28 (14)	24.01 (12)	27.66 (5)	24.21 (1)	21.33 (4)	27.43 (7)	25.27 (2)	32.94 (14)	29.17 (22)	29.19 (2)	24.77 (6)	25.96 (4)	25.19 (3)	28.76 (5)
Vitispirane II	2.45 (12)	1.68 (6)	2.07 (12)	1.72 (7)	1.52 (10)	2.78 (3)	2.39 (6)	2.84 (5)	1.66 (6)	2.04 (10)	1.47 (8)	1.27 (1)	2.30 (6)	0.14 (2)
β-Cyclocitral	18.22 (4)	18.06 (5)	16.25 (11)	15.86 (5)	13.61 (8)	22.78 (11)	17.74 (13)	22.83 (8)	22.05 (27)	22.10 (3)	18.50 (5)	17.13 (4)	18.30 (1)	17.78 (4)
TDN	21.36 (6)	21.10 (6)	16.14 (5)	7.13 (5)	11.69 (1)	17.60 (5)	14.29 (3)	28.64 (1)	22.73 (3)	22.27 (2)	19.10 (3)	22.14 (7)	19.16 (5)	17.93 (2)
β-Damascenone	79.40 (11)	70.27 (9)	69.90 (6)	64.27 (2)	59.75 (2)	81.81 (1)	79.18 (11)	94.45 (9)	91.92 (10)	91.46 (10)	91.45 (4)	91.12 (10)	85.06 (5)	81.04 (10)
Geranyl acetone	100.31 (15)	98.70 (5)	97.36 (1)	95.11 (7)	93.90 (1)	113.64 (13)	105.23 (3)	106.69 (15)	97.70 (5)	90.86 (15)	89.89 (13)	90.13 (9)	96.69 (7)	94.47 (6)
β-Ionone	0.65 (5)	0.41 (13)	0.62 (12)	0.39 (9)	0.37 (11)	0.30 (3)	0.23 (2)	0.19 (5)	0.28 (9)	0.05 (8)	0.06 (10)	0.12 (2)	0.32 (4)	0.26 (6)
**Alcohols**														
Butan-2-ol	1.21 (14)	3.19 (15)	4.07 (13)	1.61 (8)	3.51 (18)	2.87 (9)	4.02 (16)	4.57 (10)	2.65 (13)	5.18 (13)	3.84 (4)	3.93 (5)	1.68 (17)	4.40 (2)
2-Methylpropan-1-ol	0.69 (7)	0.38 (14)	0.44 (10)	0.46 (8)	0.42 (4)	0.68 (11)	0.50 (5)	0.68 (11)	0.46 (15)	0.64 (16)	0.42 (20)	0.37 (11)	0.41 (2)	0.31 (5)
Hexan-2-ol	0.62 (8)	0.75 (9)	1.27 (18)	0.99 (4)	1.48 (6)	0.98 (2)	1.18 (14)	0.90 (19)	0.58 (7)	2.09 (9)	0.94 (7)	0.93 (21)	0.47 (1)	0.73 (11)
3-Methylbutan-1-ol	192555.32 (4)	246398.50 (11)	299793.26 (4)	304279.99 (9)	275563.73 (10)	307420.94 (15)	341970.29 (2)	280499.14 (7)	392224.07 (9)	329407.10 (17)	366199.84 (20)	321330.53 (3)	250885.52 (9)	311907.84 (5)
Hexan-1-ol	232.04 (12)	167.21 (7)	162.27 (13)	87.14 (1)	188.51 (20)	121.36 (11)	127.14 (4)	251.43 (20)	204.45 (12)	234.86 (15)	244.59 (3)	199.11 (2)	95.76 (5)	147.51 (7)
(*E*)-3-Hexen-1-ol	141.52 (11)	149.98 (3)	138.11 (11)	99.93 (5)	61.44 (5)	131.61 (10)	117.10 (7)	78.27 (9)	43.47 (16)	53.69 (12)	38.63 (7)	27.26 (3)	134.12 (7)	138.01 (8)
(*Z*)-3-Hexen-1-ol	41.57 (6)	104.27 (11)	30.38 (4)	106.14 (9)	110.62 (16)	61.62 (17)	58.77 (6)	53.73 (14)	99.27 (13)	57.00 (3)	49.81 (8)	78.71 (4)	104.46 (4)	65.80 (10)
2-Ethylhexan-1-ol	371.06 (15)	330.55 (5)	325.18 (4)	325.68 (13)	325.73 (9)	320.76 (4)	366.82 (19)	329.96 (9)	279.25 (25)	239.63 (9)	235.20 (4)	239.10 (9)	275.33 (2)	323.56 (9)
(R,S)-Butan 2,3-diol	33.16 (10)	18.38 (9)	20.51 (2)	33.04 (1)	21.82 (16)	21.37 (12)	20.38 (11)	28.96 (4)	25.27 (17)	27.75 (10)	23.55 (5)	25.01 (6)	22.98 (7)	22.99 (10)
(R,R)-Butan-2,3-diol isomer	52.05 (16)	170.33 (7)	188.67 (2)	139.38 (5)	142.66 (9)	150.61 (7)	168.06 (9)	107.51 (4)	162.21 (11)	160.56 (13)	170.32 (4)	164.20 (13)	143.85 (1)	151.84 (6)
Decan-1-ol	4.67 (19)	3.42 (6)	1.91 (3)	1.22 (6)	1.22 (4)	3.95 (5)	1.27 (8)	5.01 (6)	1.46 (9)	4.54 (12)	0.93 (3)	3.58 (4)	3.23 (5)	3.70 (5)

Benzyl alcohol	2836.56 (18)	2522.45 (8)	2791.56 (7)	2522.18 (8)	2971.15 (5)	2432.73 (3)	2118.30 (15)	4361.97 (10)	2836.63 (9)	4182.39 (10)	6874.64 (1)	2881.23 (6)	2118.61 (6)	2836.41 (10)
2-Phenyethyl alcohol	11361.45 (20)	16027.96 (1)	19976.66 (19)	15938.22 (6)	28950.59 (6)	13919.09 (5)	15085.95 (17)	14412.71 (16)	28681.20 (19)	32988.56 (8)	27784.04 (12)	18720.12 (6)	10913.04 (2)	10867.96 (4)
**Sulphur compound**														
Ethyl 3-(methylthio)propionate	0.45 (12)	0.53 (11)	- -	- -	- -	0.62 (8)	1.28 (11)	1.25 (2)	0.21 (27)	- -	- -	- -	0.84 (7)	0.36 (9)
**Esters**														
Ethyl butanoate	235.67 (10)	280.84 (4)	370.20 (8)	594.91 (7)	96.60 (14)	101.13 (12)	101.23 (16)	190.70 (3)	191.02 (15)	280.65 (3)	321.61 (5)	319.04 (12)	314.78 (14)	311.60 (12)
Ethyl 3-methylbutanoate	18.01 (14)	17.26 (17)	16.20 (8)	11.56 (6)	10.52 (6)	6.20 (13)	2.67 (7)	20.04 (6)	15.72 (17)	13.43 (16)	11.29 (13)	7.29 (15)	5.84 (8)	3.09 (4)
Isoamyl acetate	228.79 (10)	188.07 (6)	183.81 (7)	165.77 (2)	173.57 (4)	171.78 (14)	146.86 (5)	154.03 (3)	150.01 (5)	137.09 (5)	136.71 (13)	123.85 (19)	114.71 (7)	106.96 (9)
Ethyl hexanoate	5166.14 (16)	6332.86 (18)	7005.77 (7)	6781.23 (2)	8800.53 (1)	4941.53 (3)	7050.77 (1)	5839.24 (10)	6288.00 (12)	7050.42 (4)	7768.48 (6)	7544.06 (16)	4268.70 (7)	7678.60 (6)
Hexyl acetate	69.34 (8)	70.50 (1)	69.26 (11)	69.50 (6)	70.25 (4)	68.60 (10)	68.61 (7)	69.00 (7)	68.94 (3)	69.31 (5)	71.03 (5)	69.80 (9)	68.80 (9)	69.83 (8)
Ethyl pyruvate	1.44 (10)	1.57 (10)	1.59 (4)	1.49 (8)	2.01 (2)	0.71 (7)	0.87 (3)	1.99 (3)	1.34 (8)	2.01 (9)	1.98 (1)	1.77 (10)	0.72 (2)	1.46 (6)
Ethyl lactate	15831.91 (6)	12601.54 (5)	12870.45 (4)	15203.98 (16)	17626.71 (7)	9684.82 (2)	12915.26 (2)	14979.22 (18)	14665.14 (2)	8007.62 (11)	11389.92 (5)	14755.24 (4)	12421.85 (5)	15562.96 (9)
Ethyl octanoate	1110.06 (1)	1120.56 (10)	914.08 (10)	929.47 (8)	986.13 (8)	1156.81 (2)	923.26 (15)	1116.25 (2)	1075.90 (2)	1160.79 (1)	1026.24 (12)	1241.87 (2)	936.65 (12)	1403.44 (12)
Ethyl 3-hydroxybutanoate	90.62 (12)	95.71 (9)	96.00 (6)	96.86 (13)	98.33 (7)	96.35 (13)	54.57 (4)	99.47 (11)	54.68 (16)	99.36 (18)	144.68 (5)	96.53 (9)	56.02 (4)	69.84 (2)
Ethyl decanoate	7413.04 (11)	7637.34 (20)	4585.03 (6)	2118.52 (5)	4721.11 (4)	5348.97 (11)	4586.30 (15)	7771.89 (13)	2253.22 (7)	6066.81 (4)	3329.95 (2)	5707.95 (7)	8445.04 (7)	70.68 (13)
Diethyl succinate	24293.34 (1)	20255.24 (6)	20703.95 (15)	25639.59 (6)	33716.16 (12)	116275.81 (8)	140505.35 (1)	57496.60 (8)	67368.07 (1)	66021.85 (9)	111340.04 (1)	29677.58 (9)	165874.21 (2)	156658.19 (2)
Ethyl 3-hydroxyhexanoate	0.13 (18)	0.30 (12)	0.41 (8)	0.35 (18)	0.49 (5)	0.13 (12)	0.22 (9)	0.30 (9)	0.28 (10)	0.50 (19)	0.56 (14)	0.46 (4)	0.09 (8)	0.13 (3)
Ethyl 9-decenoate	9.79 (11)	7.02 (13)	10.85 (6)	11.25 (12)	16.11 (1)	4.46 (2)	6.89 (6)	14.63 (7)	12.72 (13)	19.16 (7)	18.19 (10)	12.34 (10)	3.99 (8)	8.25 (2)
Ethyl benzeneacetate	1.77 (6)	1.62 (6)	1.42 (8)	1.74 (7)	1.26 (6)	1.75 (7)	1.26 (5)	2.45 (9)	1.64 (3)	1.92 (12)	0.93 (6)	2.73 (12)	1.24 (8)	1.67 (1)
Ethyl dodecanoate	969.67 (8)	970.66 (4)	522.61 (5)	521.25 (9)	523.10 (12)	523.35 (2)	635.78 (18)	994.66 (7)	949.73 (20)	814.92 (15)	590.76 (4)	814.98 (5)	949.87 (7)	994.72 (8)
Ethyl salicylate	0.64 (10)	1.26 (2)	2.13 (15)	1.66 (6)	1.60 (11)	0.25 (10)	0.56 (8)	0.78 (2)	0.73 (1)	0.85 (13)	1.15 (2)	2.42 (6)	0.28 (9)	0.31 (5)
Benzyl butanoate	16.03 (13)	15.58 (6)	11.59 (7)	6.17 (3)	16.57 (3)	11.27 (5)	5.60 (1)	11.80 (12)	10.46 (9)	12.47 (4)	17.24 (2)	13.40 (4)	15.09 (8)	12.40 (2)
Ethyl 2-phenylacetate	2767.21 (13)	2385.28 (16)	3216.52 (18)	2878.96 (10)	4090.54 (16)	2385.32 (6)	2071.60 (6)	2699.68 (10)	3372.47 (7)	2744.34 (5)	1846.90 (6)	3215.02 (10)	1867.50 (6)	1916.46 (8)
Diethyl tartarate	0.42 (8)	0.56 (4)	0.47 (20)	0.69 (4)	0.32 (8)	0.61 (2)	1.15 (15)	1.20 (5)	0.19 (20)	0.32 (2)	0.65 (9)	0.96 (1)	0.78 (3)	0.36 (10)
Ethyl succinate	142.22 (13)	231.64 (6)	97.34 (8)	141.94 (9)	97.32 (2)	142.05 (4)	83.88 (7)	141.99 (5)	146.36 (16)	142.16 (14)	142.11 (10)	141.95 (12)	97.26 (8)	142.06 (13)
Ethyl citrate	0.64 (10)	1.26 (22)	2.13 (15)	1.66 (6)	1.60 (11)	0.25 (10)	0.56 (8)	0.78 (2)	0.73 (10)	0.85 (13)	1.15 (2)	2.42 (6)	0.28 (19)	0.31 (5)
**Acids**														
Acetic acid	4899.25 (2)	5482.34 (8)	5078.51 (5)	6469.47 (8)	5841.17 (5)	5078.70 (2)	7456.73 (9)	7322.12 (2)	5527.26 (5)	6783.81 (10)	7546.18 (8)	8443.80 (7)	3463.10 (3)	5347.98 (3)
2-Methylpropanoic acid	1.49 (9)	1.66 (4)	1.55 (9)	1.62 (4)	1.19 (2)	0.71 (8)	0.55 (5)	0.53 (10)	1.00 (10)	0.93 (13)	0.97 (5)	1.03 (4)	0.94 (9)	0.90 (8)
Butanoic acid	1.81 (6)	2.09 (9)	2.19 (2)	2.12 (3)	2.16 (3)	2.10 (8)	2.19 (2)	0.88 (15)	0.85 (8)	0.97 (4)	0.97 (3)	1.30 (6)	1.66 (7)	1.13 (3)
3-Methylbutanoic acid	4.72 (7)	4.71 (9)	4.87 (9)	4.81 (7)	4.81 (11)	4.80 (9)	4.82 (5)	4.80 (6)	4.82 (3)	4.80 (7)	4.81 (9)	4.84 (7)	8.65 (4)	4.91 (6)
Hexanoic acid	6698.73 (9)	8448.68 (9)	6878.15 (3)	10288.46 (4)	9615.20 (9)	10736.91 (7)	10961.23 (2)	11006.24 (2)	8942.17 (2)	9570.25 (7)	9615.28 (3)	12441.90 (7)	9839.86 (5)	11813.73 (2)
2-Ethylhexanoic acid	2.06 (10)	1.99 (4)	2.03 (4)	1.82 (14)	2.45 (8)	0.92 (5)	0.60 (2)	2.22 (6)	1.51 (7)	3.98 (12)	3.04 (2)	4.92 (19)	0.58 (2)	1.47 (4)
Octanoic acid	2763.31 (3)	3660.45 (1)	4558.10 (7)	2314.59 (3)	2314.50 (6)	3660.82 (3)	2763.44 (6)	1865.68 (3)	1417.36 (8)	2763.22 (5)	1865.86 (11)	2763.44 (6)	2314.43 (8)	1865.84 (3)
Decanoic acid	2342.77 (9)	2791.44 (4)	2791.75 (4)	2791.35 (10)	1894.28 (6)	1894.32 (2)	996.61 (4)	548.13 (6)	548.24 (6)	996.73 (9)	996.71 (7)	548.21 (5)	1445.29 (3)	1445.48 (4)
Undecylic acid	0.18 (1)	0.21 (7)	0.19 (15)	0.23 (13)	0.24 (6)	0.21 (9)	0.19 (3)	0.24 (7)	0.21 (5)	0.25 (17)	0.28 (9)	0.29 (7)	0.31 (10)	0.25 (9)

**Acetals**																												
1,1-Diethoxyethane	84.00	(8)	94.31	(6)	96.88	(2)	118.12	(5)	125.71	(7)	94.68	(5)	130.61	(2)	91.22	(3)	91.87	(3)	101.28	(3)	128.56	(3)	137.77	(2)	139.24	(7)	171.24	(2)
Cis-dioxane	97.91	(4)	101.02	(5)	127.72	(4)	150.95	(3)	175.38	(10)	98.06	(9)	139.90	(7)	140.15	(4)	142.11	(9)	163.13	(6)	193.43	(2)	223.15	(6)	97.09	(6)	129.80	(9)
Cis-dioxolane	45.96	(17)	50.62	(2)	56.91	(10)	67.37	(11)	88.11	(9)	46.69	(3)	60.09	(9)	51.40	(7)	56.16	(4)	61.65	(3)	67.26	(6)	86.37	(9)	45.29	(4)	48.22	(4)
Trans-dioxane	42.46	(14)	42.91	(2)	42.80	9)	43.74	(9)	45.83	(11)	41.63	(2)	46.52	(6)	41.81	(2)	41.97	(2)	44.70	(6)	47.78	(9)	53.85	(6)	42.02	(2)	42.75	(3)
**Furanic compounds**																												
2-Acetylfuran	1.35	(5)	2.01	(3)	2.44	(4)	2.63	(3)	2.84	(4)	1.01	(8)	1.20	(5)	1.26	(3)	1.18	(7)	1.50	(4)	1.76	(6)	1.22	(9)	0.74	(5)	1.02	(3)
2-Furfural	174.39	(8)	215.85	(8)	217.72	(10)	260.59	(2)	307.28	(2)	219.28	(3)	241.78	(3)	210.33	(2)	246.29	(6)	268.35	(2)	300.07	(2)	322.29	(2)	192.26	(1)	281.98	(8)
1-(2-Furyl)-1-propanone	1.86	(2)	4.07	(7)	2.77	(9)	4.56	(9)	4.02	(5)	0.95	(4)	1.41	(6)	1.23	(9)	1.29	(10)	2.21	(8)	4.49	(19)	2.53	(11)	0.98	(8)	1.11	(4)
5-Methyl-2-furfural	2.25	(3)	4.57	(2)	5.74	(6)	3.52	(5)	6.65	(2)	4.95	(6)	6.52	(3)	3.10	(2)	2.77	(7)	7.15	(3)	6.86	(3)	7.01	(9)	2.97	(6)	3.97	(3)
Ethyl 2-furoate	8.67	(7)	8.96	(7)	8.84	(3)	13.14	(6)	13.42	(3)	22.30	(2)	35.64	(7)	35.86	(10)	53.77	(6)	67.07	(17)	89.60	(14)	103.09	(7)	17.57	(9)	31.30	(6)
5-Ethoxymethyl-2-furfural	0.96	(10)	1.15	(6)	1.66	(4)	1.48	(4)	1.51	(5)	0.96	(3)	1.56	(9)	1.09	(3)	1.29	(7)	1.69	(5)	2.58	(3)	2.81	(6)	1.20	(5)	1.74	(3)
5-Hydroxymethyl-2-furfural	41.87	(9)	41.96	(6)	50.50	(2)	56.81	(3)	91.42	(10)	29.81	(2)	63.66	(2)	42.75	(2)	44.80	(3)	54.87	(2)	79.81	(6)	101.55	(3)	42.06	(9)	55.63	(4)
**Lactones**																												
Butyrolactone	-	-	-	-	1.38	(2)	1.39	(6)	1.29	(4)	-	-	1.18	(5)	0.50	(9)	0.57	(6)	1.14	(10)	1.92	(3)	1.26	(7)	-	-	0.74	(14)
γ-Hexalactone	116.97	(4)	116.99	(19)	121.52	(14)	125.97	(7)	135.07	(12)	-	-	112.38	(11)	116.75	(6)	116.95	(9)	126.10	(8)	135.16	(17)	143.94	(12)	148.23	(14)	149.40	(8)
γ-Octalactone	112.45	(3)	115.29	(8)	116.96	(16)	112.38	(2)	127.89	(4)	-	-	49.48	(18)	-	-	-	-	112.65	(16)	121.51	(16)	139.39	(7)	133.01	(1)	134.89	(7)
γ-Decalactone	-	-	175.47	(16)	179.64	(13)	184.11	(1)	188.75	(11)	-	-	175.06	(17)	175.50	(19)	177.37	(9)	177.85	(13)	179.73	(9)	184.18	(6)	188.66	(2)	193.14	(1)
(*Z*)-Whiskylactone	-	-	-	-	60.37	(2)	94.57	(3)	97.34	(3)	63.45	(3)	64.10	(9)	63.34	(2)	65.29	(3)	74.40	(2)	102.45	(2)	119.39	(3)	89.87	(11)	104.67	(3)
γ-Dodecalactone	-	-	-	-	0.05	(5)	0.16	(8)	0.19	(1)	-	-	0.29	(17)	-	-	-	-	0.05	(5)	0.10	(19)	0.15	(1)	-	-	0.28	(13)
**Volatile phenols**																												
2-Phenoxyethanol	0.55	(10)	1.01	(2)	0.39	(17)	0.34	(12)	0.50	(8)	0.33	(1)	0.26	(12)	0.26	(17)	0.17	(4)	0.23	(7)	0.26	(2)	0.19	(7)	0.23	(3)	0.72	(4)
Eugenol	4.55	(8)	9.40	(14)	9.19	(11)	9.96	(13)	12.01	(8)	13.97	(14)	18.09	(14)	18.72	(4)	18.13	(6)	27.45	(9)	27.57	(3)	31.57	(3)	27.27	(2)	27.43	(3)
Vanillin	9.43	(3)	13.80	(15)	14.11	(9)	13.56	(14)	18.42	(3)	13.64	(8)	14.49	(9)	-	-	-	-	27.72	(3)	31.64	(4)	36.61	(7)	-	-	18.16	(2)
Methyl vanillate	-	-	-	-	1.36	(5)	0.92	(8)	1.12	(6)	0.51	(8)	0.87	(9)	-	-	-	-	0.09	(11)	0.37	(2)	0.44	(5)	0.51	(5)	0.47	(8)

[1] YB - blend wine - years, average age; [2] Y - vintage wines - age expressed in years; - : not detected; TDN: 1,2-Dihydro-1,1,6-trimethylnaphthalene
